# Bayesian Cue Integration as a Developmental Outcome of Reward Mediated Learning

**DOI:** 10.1371/journal.pone.0021575

**Published:** 2011-07-05

**Authors:** Thomas H. Weisswange, Constantin A. Rothkopf, Tobias Rodemann, Jochen Triesch

**Affiliations:** 1 Frankfurt Institute for Advanced Studies, Frankfurt, Germany; 2 Honda Research Institute Europe GmbH, Offenbach, Germany; University of Sheffield, United Kingdom

## Abstract

Average human behavior in cue combination tasks is well predicted by Bayesian inference models. As this capability is acquired over developmental timescales, the question arises, how it is learned. Here we investigated whether reward dependent learning, that is well established at the computational, behavioral, and neuronal levels, could contribute to this development. It is shown that a model free reinforcement learning algorithm can indeed learn to do cue integration, i.e. weight uncertain cues according to their respective reliabilities and even do so if reliabilities are changing. We also consider the case of causal inference where multimodal signals can originate from one or multiple separate objects and should not always be integrated. In this case, the learner is shown to develop a behavior that is closest to Bayesian model averaging. We conclude that reward mediated learning could be a driving force for the development of cue integration and causal inference.

## Introduction

Empirical studies have provided convincing evidence that humans combine sensory signals into percepts so as to reduce the uncertainty about their causes. Such studies, as reviewed in [1]–[6], have commonly used the cue integration paradigm, in which human observers are asked to infer a certain quantity based on the observations of a bi– or multisensory signal. These experiments may use stimuli across modalities such as in the judgment of the position of an object based on visual and auditory cues [7], [8] or object size given visual and haptic cues [9]. Similarly, experiments have considered cues within the same modality as in inferring surface slant from stereo and texture cues [10] or depth from texture and motion cues [11]. The overwhelming majority of these studies has shown that humans combine these cues by weighting them according to their relative reliabilities.

This empirical behavior has been modeled as inference of the most likely cause of the observation given the sensory cues and prior knowledge using the Bayesian framework. Bayesian inference represents the uncertainty about parameters in the inferential task explicitly as probability distributions. The variance of the distribution resulting from optimally combining the different sources of information is smaller than that of the individual sensory distributions which reflects the reduction in uncertainty.

The Bayesian inference framework has recently been extended to cases in which the observed sensory signals are caused by one of two different scene configurations [12], [13]. As an example, consider the case in which a visual and an auditory cue are sensed. Whether these cues should be integrated or not may depend on the assumptions about the causes for these signals. If two separate objects caused them, they should not be combined, whereas if the two signals are likely to be caused by a single object in the scene, they should be integrated. By representing the uncertainty about the two possible scene configurations, the Bayesian framework can be used to compute a posterior probability distribution over the two scene layouts, a process that has been termed causal inference [13], [14]. Instead of inferring the scene layout, the task may be to compute a posterior probability of the positions of the signal source by weighting the likelihoods of the two scene layouts. While model selection is the optimal strategy for the former task, the latter task favors Bayesian model averaging. In most experimental setups however, it is difficult to distinguish which of these strategies best matches human behaviour or whether humans use a fixed single strategy at all [15].

Despite the aforementioned successes in applying the Bayesian framework to sensory perception, a wide variety of questions remain open at the computational, algorithmic, and implementation levels, see e.g. [6], [16]. The most central issue is how cue integration and causal inference are learned on developmental timescales [17]–[20]. A recent empirical study by Gori and colleagues [17] showed that children under 10 years of age did not integrate cues by taking their uncertainties into account. Instead, these children were shown to almost exclusively use haptic cues in a size discrimination task and visual cues in an orientation discrimination task independently of their reliability. This suggests that the abilities for cue integration and causal inference are acquired through development. This result is particularly challenging for some current theoretical models at the implementational level suggesting that cue integration is mediated simply by the Poisson-like trial to trial variability in neuronal populations [21], [22]. These so called probabilistic population codes were shown to be able to integrate probability distributions, represented by probabilistic activity within specific neuronal populations, using simple biologically plausible computations. However, it is unclear how such a mechanism could be learned over developmental timescales. It is an open question then, why infants and children do not integrate cues optimally and how learning could proceed [16].

Further evidence for the role of learning in the development of sensory integration and causal inference comes from a study by Putzar et al. [23] in which the authors show that early deprivation of one modality during the first month of life impaired multisensory integration including this sense even after complete recovery (see also [24]). This matches neurophysiological findings in cats and monkeys suggesting a critical period of high plasticity and large changes in receptive fields of multisensory neurons during early development [25], [26]. Neurons in superior colliculus of newborn kittens and monkeys show little or no multisensory responses, but the number of multisensory neurons grows and their tuning gets sharper with age.

There is also initial evidence, that the mechanisms involved in causal inference are not fully developed at birth, at least in cats [27]. Cats were raised in an artificial environment, in which auditory and visual signals were always shown at the same time but at differing spatial positions. Subsequent behavioral as well as neurophysiological tests revealed that the animals did not integrate multisensory stimuli from a common location, as seen in animals raised in natural environments, but instead integrated only signals with the distinct spatial separation present in the artificial environment. For similar results in owls see [28].

Based on these results, the present study asks, whether cue integration and causal inference in sensory perception could develop mediated by reward dependent learning. There are ample data demonstrating reward dependent learning related to orienting movements [29]–[31]. Furthermore, there has been considerable work relating this type of learning to theoretical models of reinforcement learning (RL), see e.g. [32]. Various studies were able to localize areas in the human and monkey brain potentially implementing RL mechanisms by looking for correlations between RL model variables and single cell [32], [33] or BOLD activities [34]–[40]. Those studies show the relevance of RL for learning in many different tasks and environments both on the behavioral as well as the computational level. Thus it is interesting to also consider it as one potential driving force, on the computational level, for the development of cue integration and causal inference.

## Results

**Figure 1:**
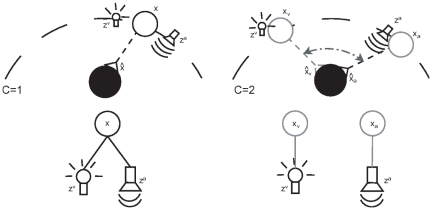
Scene layout of orienting tasks and generative models. Top: Sketch of the orienting task used in this study. The learner receives an auditory (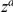) and a visual (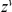) signal, which are probabilistically related to the true position 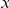. The task is to orient towards this true position. Left: A case where the visual and auditory signals have a common cause. Right: The signals originate from different locations. Bottom: The generative models for the task. The noisy sensory signals (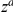 and 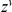) are either generated by a single (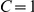) or two independent causes (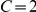) having different spatial positions.

We use a multimodal localization task similar to the one used by Neil and colleagues [20] and Körding et al [13] (see Fig. 1 for a schematic depiction). The learner obtains noisy visual and auditory signals and carries out horizontal orienting movements, obtaining a varying amount of reward dependending on the accuracy of the movement (see Methods). We interpret the reward as an intrinsic signal for bringing a relevant stimulus into the center of attention.

The agent learns to solve this task based only on its sensory inputs, orienting actions, and observed rewards. To this end, it learns to predict how much reward to expect when performing each action in a given situation. The learner represents its reward estimates for particular state and action pairs as so called Q-values [41]. Support for the representation of such variables in the human and monkey brain comes from several studies [33], [42]. In our case this Q-function is approximated by a neural network (see Methods). Based on these reward expectations, the agent will probabilistically pick an action using a softmax function, which also has been shown to match human action selection for some tasks [43], [44]. The reward prediction of the winning action will be adapted depending on the difference between predicted and obtained reward by changing all synaptic weights via a gradient descent learning algorithm (see Methods).

In the following we will test this model on cue integration and causal inference tasks and compare it to human behavior and four different Bayesian models.

### Cue Integration

**Figure 2:**
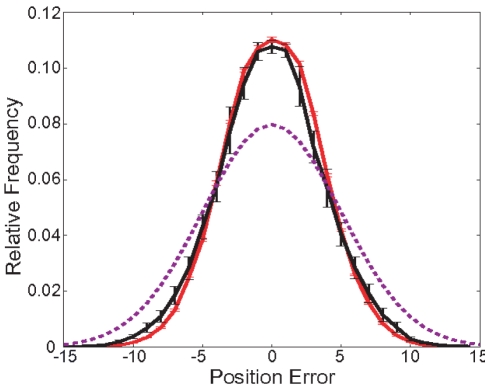
Distribution of position estimation errors. The distribution of errors over 100,000 orienting actions carried out by the RL model after 10 runs with each 100,000 training steps (black), compared with Bayesian optimal integration (red) and the best single cue predictions (dashed) for a single audio-visual object. Errorbars show standart deviation of 10 runs. (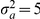, 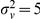).

We start with a simple cue integration paradigm, where noisy auditory and visual signals from a common source have to be combined. If the noise of the two cues is independent, the variance of the error produced by optimally integrating the two stimuli is always smaller or equal to the error variance resulting from using either cue alone. Figure 2 shows the distribution of errors the RL based model produces after training. This result matches well with the predictions of the optimal Bayesian model for this situation.

To compare the model with human behavior, we test the fully trained model on a two-alternative forced choice (2-AFC) task. This task allows us to test the behavior of the learner for changes in relative reliabilities between the cues. The setup is similar to the one used by Ernst and Banks [9], where human subjects were asked to perform a 2-AFC visuo-haptic size discrimination task. Ernst and Banks could show that in this task the point of subjective equality (PSE) of adults is well predicted by Bayesian cue integration and shifts when additional visual noise is introduced.

**Figure 3:**
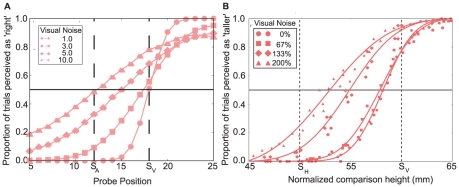
Psychometric curves for 2-AFC task in comparison to human psychophysics. A: Psychometric curves for the proportion of ‘right’-actions in an audio-visual 2-AFC position estimation task. The input positions of the standard were mismatched, with the auditory signal positioned at 12 and the visual signal at 18. Probe inputs were matched and tested 1,000 times at each position (0–30). The point at which the curves cross the black vertical line is the PSE. The curves differ in the variance of the visual noise (see legend), auditory noise was kept constant with 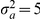. B: Plot using data from a psychophysical experiment by Ernst and Banks [9]. They used a visuo-haptic 2-AFC size discrimination task and count the proportion of ‘taller’-actions. The standard inputs were mismatched (haptic at 50, visual at 60), probe inputs were matched and varied between 45 and 65. The visual reliability was varied by adding external noise to the display.

The first input to the agent is the size of a standard bar with constant position, the second is the size of a probe which varies and is to be estimated as ‘left’ or ‘right’ of the standard (respectively ‘taller’ or ‘smaller’ in [9]). Both stimuli are bimodal, but for the probe the cues are always consistent, whereas for the standard they are set to be in conflict with each other. Figure 3A shows the proportion of ‘right’ estimates for all possible positions of the probe based on the decisions taken by the reinforcement learner after training as psychometric curves. Each curve represents training and testing with a different visual noise variance. We can compare it with the data from of Ernst and Banks [9] (Figure 3B) which is reproduced in our Figure 3B. It shows the equivalent data for the average of four human subjects. Both plots show a similar pattern in that the psychometric curves get steeper and the PSE moves more towards the visual stimulus position for decreasing visual noise levels. Figure S2 compares the PSEs of the RL model (crosses) with that of the optimal Bayesian observer (circles) for different visual reliabilities. It can be seen that they match quite well, as was true for the human subjects in [9] (Fig 3c in their paper). Note that there is variability in both the PSEs of the learner and the Bayesian observer due to the limited number of test stimuli.

### Causal Inference

In the following tasks we will add a second layer of complexity to the task by randomly presenting trials that were generated by different scene layouts, i.e. under either the common or the separate cue condition. We will compare our learned model with four Bayesian observers. One observer always integrates the information from the two stimuli (we will call that one AI). A second always acts as if both stimuli originate from different objects and discards information from the less reliable modality (“Never Integrating” – NI). A third, more advanced, observer computes the probability of one vs. two objects in each trial and uses the optimal action for the more probable model (“Model Selection” – MS). The fully optimal fourth observer though makes use of all information available by selecting an action under the weighted evidence for each generative model, with the weights proportional to the respective probabilities (“Model Averaging” – MA). All Bayesian observers, contrary to the RL model, have explicit knowledge of position priors, sensory noise distributions and the reward rule. The mathematical formulation of these decision rules as well as the reward expectations of the observer models can be found in the Methods section.

**Figure 4:**
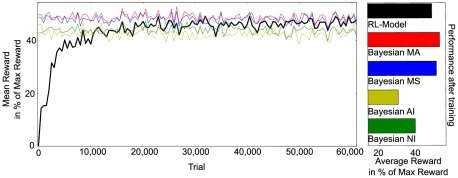
Performance of the RL model and Bayesian observers for a single output. Reward obtained by the learner when choosing the action with highest predicted reward (black) compared to the different Bayesian observers. Signals can originate from one or two objects. Left: Change of performance during learning. Each data point is the sliding average of 1000 trials. Right: Barplot of the mean reward over 100,000 trials after learning. Standard error of the means is smaller than 0.5% for all bars. (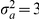, 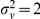).

To show the learning process of the RL learner, we can look at the development of the potential reward received with a greedy policy (always selecting the action which predicts the highest reward). In Figure 4 one can see that the average reward earned by the learner increases until it reaches a level similar to what the MA and MS models show (see also Table 1 1A). Comparing it with the simpler instances of Bayesian observers, the learner is clearly better than AI and NI, that is, it implicitly incorporates the existence of two different conditions. But it is hard to tell apart the Bayesian MA and MS observers. Both are similar to the agent's performance in this task.

**Table 1:** Model performance for different set-ups.

Setup	RL-Model	Bayesian MA	Bayesian MS	Bayesian MI	Bayesian NI
1A	46.62%	47.9%	47.04%	37.87%	41.57%
1B	46.32%	47.9%	47.04%	37.87%	41.57%
1C	45.86%	47.06%	46.18%	37.28%	40.88%
2A	50.51%	51.81%	51.08%	41.63%	47.71%
2B	36.26%	37.37%	36.53%	33.87%	34.28%
2C	48.74%	50.52%	49.70%	40.32%	45.67%

Average fraction of maximum reward received in 100,000 steps after learning (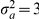, 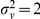) for different variation the task and the model. Results of the different Bayesian observers for comparison.

**Figure 5:**
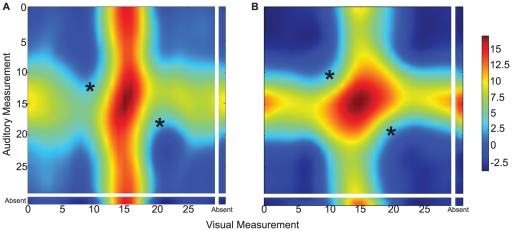
Exemplary subsections of two learned Q-value functions. Expected reward for visual signals (x-axis) and auditory signals (y-axis) for the action of orienting towards the center. Red colors represent high, blue low predicted rewards. Left: Visual cue is more reliable (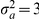, 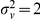); Right: Both cues have the same variance (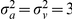). For a detailed explanation see main text.

A different way of assessing the behavior of the RL agent is to directly consider the expected total discounted reward obtainable for a particular state-action pair, the Q-values. Figure 5 shows subsections of two learned Q-value function approximations for all inputs, a given action nd two different reliability ratios. The highest reward is expected if both input signals are close together, resulting in a high probability for a single cause, and close to the target of the given action, resulting in a high probability for the action being correct (Fig. 5 center of both plots). Importantly, if the target of the given action can not possibly be a result of a weighted average of the input positions – because the cues favour both a higher or both a lower position, this action predicts little reward (Asterisks in Fig. 5). For this reason the plots show an asymmetric reward landscape. The slant of the area of highest reward (dark red) depends on the relative reliability of the two cues, as can be seen when comparing A and B in Fig. 5. The left plot is a result of inputs with a higher reliability in the visual modality, therefore the area of highest reward lies more along the visual axis, whereas in the right plot with equal reliabilities for both cues it lies along the diagonal exactly between the auditory and visual axis. The width of this area, as well as the maximum predicted reward, is determined by the absolute values of the reliabilities (narrower and darker red in the left plot due to higher visual reliability). A smaller reward can be expected if the cues are far apart – resulting in a high probability for two causes, but one of them is close to the action target – resulting in a high probability for the action to be correct for one of the objects (Middle of each of the four figure boundaries in Fig. 5 – the “arms” of the cross). The height of these expectations depends again on the reliability of the relevant cue.

In the experimental setup from [13] participants were asked to report in each trial both the visual as well as the auditory location of a stimulus. To mimic this condition, we change our task accordingly and add a second output population to the neural network (see Fig.S1). Each population now represents the actions for one modality (representing the arrays of buttons for the participants in [13]). The rewards and the prediction errors are computed separately (according to (3)). Table 1 2A shows the performance after learning as the sum of both rewards. The effects are similar to the previous orienting task, in that we see a performance thats is similar to the predictions of MA and MS.

**Figure 6:**
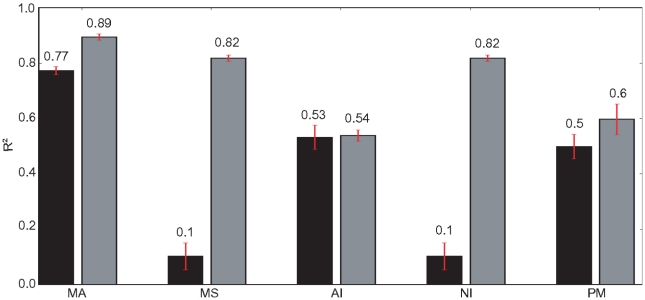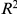 values of different observers for the RL model with selected inputs. The black and gray bars show the results for the auditory and the visual output for 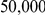 trials with inputs that differ in the predicted action between MA and MS. Mean over 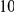 training sessions with 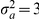, 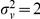, errorbars show standard deviation.

Despite changing the task it is still difficult to distinguish these two Bayesian observers (MA and MS) from each other by comparing the collected reward. A better discriminator should be the variance explained by each observer in relation to the total variance of the orienting error of our model (generalized coefficient of determination 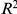 [45]). The differences between MA and MS over all inputs are nevertheless still small (Suppl. Fig.S3). Fortunately, since we have the full observer models, we can find the inputs for which the optimal actions differ between MA and MS, and then test the RL model only on those. The 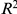 values for these inputs are shown in Fig. 6. We also include an observer which does probability matching (PM) for model selection, proposed to be the strategy used by many human subjects in a recent experiment [15]. It can be seen that the Bayesian observer with model averaging explains the error variances best for both visual and auditory output (grey and black bars). The values for the MS and NI observer are the same, because the selected inputs represent those in which MS decides to act according to the generating model with independent objects.

### Complex Uncertainty Structures

While the presented system is certain to accommodate different prior distributions of the relevant scene variable relevant for obtaining rewards (see Table 1 1C for an example with a Gaussian prior for the visual stimulus) because of the maximum total discounted reward guarantees of RL, it is interesting to see whether the system can handle different likelihood landscapes. In many real-life situations, the uncertainty of a cue depends on a number of factors. The following three experiments introduce behaviorally plausible variations in uncertainty structure and investigate how the RL agent can adjust to these.

#### Spatial Variation in Uncertainty Structure

**Figure 7:**
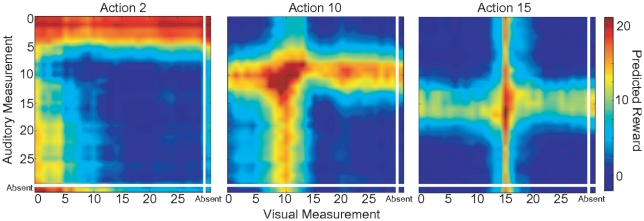
Exemplary subsections of the learned Q-value function for the foveation setup. Axes are the same as Fig. 5, but for three different actions with constant auditory reliability (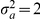) and space varying visual reliability. L-R: Actions towards a peripheral(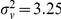), intermediate (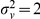) and central position (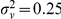).

Visual estimates of spatial location should be more accurate in the fovea than in the periphery of the visual field, given the human acuity falloff (e.g. [46], see also [47] for an example in slant angle space). Figure 7 shows the reward predictions for a set-up that mimics this observation in the task that requires a single action. The variance of the visual noise was low for stimuli in the center and increased with eccentricity, whereas auditory reliability stayed constant (Figure 7 shows results with linear increase of the variance, but similar results are reached with other functions, e.g. logarithmic decay). Training on this adapted task resulted in reward predictions dominated by the visual estimate for actions towards the center (Fig. 7 right) and dominated by audition for the outer periphery (Fig. 7 left). In between these two extremes, integration of both cues predicted the highest reward. This can also be seen in the distribution of input weights to the hidden layer (Suppl. Fig.S4). The weights from the auditory part of the input layer have similar shapes and width across all positions, whereas the visual weights get narrower towards the central positions. This shows that reward mediated learning results in behavior that varies with context within a single task, which is in accordance with predictions from a Bayesian model that explicitly takes into account context when computing the data likelihood.

#### Temporal Variation in Uncertainty Structure

**Figure 8:**
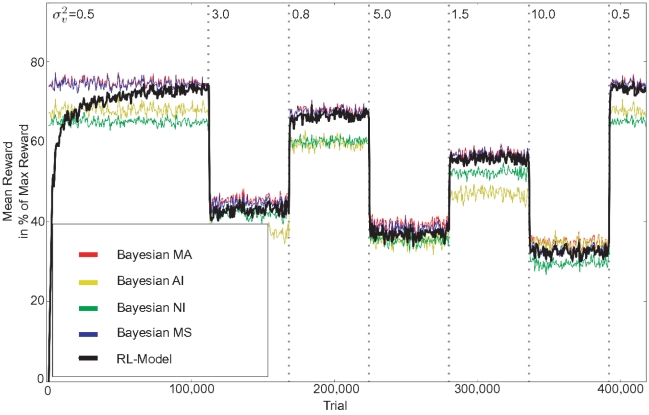
Performance of the RL Model for two outputs and temporally changing reliabilities. Reward obtained by the learner when choosing the action with highest predicted reward (black) compared to the different Bayesian observers. At each dotted vertical line visual reliability changes. Each data point is the sliding average of 1000 trials. 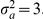

In addition to a change in noise variance across space as discussed above, in a natural environment the variance also changes over time. As an example one may consider the change in the optimal weighting of visual compared to auditory cues when stepping out of a dim room into the bright sunlight. Due to higher contrasts and thus smaller uncertainty, visual localization will gain confidence in the latter condition. To simulate such dynamics, we change the reliability of the visual cue at certain timepoints during training (Fig. 8). The network quickly adjusts to a change in visual reliability. The performance after a change point (vertical lines in Fig. 8) quickly becomes similar to the optimal predictions by the Bayesian observers. This is mostly due to the generalization abilities of the function approximation. A learner using a table with entries for every state-action-reward mapping [48] has to effectively relearn its policy with every change in conditions.

#### Shift in Uncertainty Structure

We can also adapt our settings to simulate the conditions used in the experiment by Wallace and Stein [27] to introduce mismatches in the spatial alignment of stimuli from a common object. We ask whether reinforcement mediated learning could also produce results similar to the aberrant spatial integration found in their study. Therefore we bias the auditory signal by setting the mean of its noise distribution to a value different from zero.

**Figure 9:**
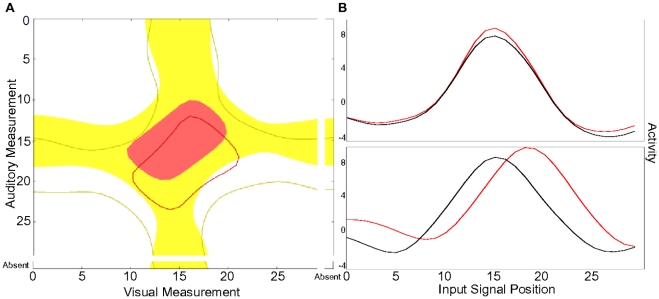
Responses of output neurons after training with auditory shift data. A: Overlay of contour plots of the Q-value functions for one action after unbiased training (filled areas) and after training with a 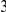-position shift in the mean of the auditory noise (empty areas). The contours include areas with predicted reward values higher than 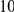 (red) and 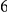 (yellow). B: Unisensory tuning curves of the same output neuron for biased (red) and unbiased conditions. The maximum visual response (top) does not change, whereas it is shifted by 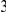 positions in the auditory domain (bottom). (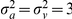).

Figure 9A shows contour plots of the Q-value function for one particular action after normal (filled) and biased training (empty). The area which favors integration (red) shifts by as many positions on the auditory axis as are introduced by the bias. The same is true for the unisensory tuning curves (Figure 9B), which were generated by plotting the response of the same output neuron to sequential single stimulation of each unisensory input neuron. These results are qualitatively similar to the ones reported by Wallace and Stein for the relationship between auditory and visual receptive fields of single neurons in cat superior colliculus.

## Discussion

The fact that cue integration in sensory inference can be well matched by Bayesian models has led to the suggestion that such computations are implemented in the brain by explicit computations with uncertainties. Accordingly, current research is looking for ways in which populations of neurons could implement Bayesian computations involving probability distributions [16], [22]. This view has led to the often implicit and sometimes explicit assumption, see e.g. [5], [49], [50], that reward dependent model free learning does not mediate this behavior. Often these investigations are accompanied by the implicit assumption, that a single algorithm has to be attributed to the brain, despite the fact that recent work by Daw and colleagues [51], [52] has shown that learning in certain tasks can be best explained by assuming that multiple learning systems implementing different algorithms are working together in the brain (see also [53]–[56]). Currently it is unclear how cue integration and causal inference are learned over developmental timescales, as experiments with both children and animals suggest that cue integration abilities develop over time [17]–[20], [23], [27], [57], [58].

Bayesian models of cue integration have been extended to cases in which there is uncertainty about the underlying scene configuration that generated the sensory stimuli [12], [13]. In this case of causal inference the observer has to judge how likely one of the possible scene configurations may have caused the observed sensory signals. There are two main ways how this could be done. Either the observer decides on the more likely scene layout and then interprets the signals according to this layout (model selection), or the positions of the objects in the scene are judged according to the likely positions according to both models and then are weighted by how much evidence there is for either layout (model averaging). Current empirical evidence is inconclusive whether human performance in such tasks is better explained by model selection, model averaging, probability matching or all of those [13], [15], [59], [60]. Similarly, it is unclear, whether any additional contextual or task effects might affect the strategy used.

Here we investigated whether a reward based model free learner using function approximation is able to learn an orienting task requiring integration of cues. Furthermore, as the cues could either originate from different sources or a single one, it was necessary to learn not to combine the estimates always, but to take into account that at larger separations of the two cues it is more likely that they originate from two different sources. The learner was given two audio-visual orienting tasks to solve. In the first task the learner was rewarded for orienting towards either one of the two stimuli, whereas in the second task the learner was rewarded separately for judging both the position of the visual and the auditory sources.

Under both task conditions the learner was able to carry out actions that combined cues according to their relative reliabilities. The reward obtained when following the reinforcement learner is higher than that obtained by the Bayesian learners that always or never integrate. It was also shown that the behavior of the RL model best matches that of a MA observer. This does not necessarily mean that humans always use MA, but shows the general ability of RL to approach optimal behavior. A recent paper by Wozny and colleagues [15] found evidence for a majority of subjects acting most similar to probability matching (at the causal inference level), but also a significant number of people that were better fit by model averaging. Further research is needed to clarify whether this is generally true or depends on additional parameters.

We could also show that the RL approach is able to deal with more complex uncertainty structures in the input. Here, the uncertainties are implicitly represented in the function approximation scheme of the value functions. Arguably, representing only uncertainties that are relevant for obtaining rewards is more economical than representing all potential distributions over all available scene variables. Indeed, here the distributions over sensory cues given relevant scene variables were not provided beforehand to the system, as is common in the Bayesian cue integration and causal inference setting. The proposed model was able to also perform similarly to the Bayesian predictions when the data likelihood was variable in time or space, when using non-uniform priors, and for changes in the causal structure. Humans were shown to be able to rapidly adapt to changes in cue reliability [61]–[63] and causal layout [64]. Although we do not want to claim that this is necessarily mediated by reward for the very early adaptation, we show the potential of RL-mechanisms to react to those changes. It would also be interesting to test children for the developmental aspects of such rapid re-weighting [65], but more experiments will be needed to clarify those results.

One feature that is missing in our approach is temporal relations between signals, which in a natural environment provide an important cue for causal inference (e.g. [66]). It was shown that this influence is also plastic in children [20] and in adults [67], so it would be interesting to see how reward mediated learning deals with the incorporation of temporal information. The TD–learning framework is in principle able to deal with delayed rewards. This question will be addressed by future work.

All learning was done with immediate reward feedback to individual actions using learning rules that have been well established in conjunction with reward related learning and orienting movements [31], [32]. We are aware that using gradient descent learning to update the weights of the neural network could be considered problematic for a neural implementation [68]. In the recent past, attempts were made to relate this kind of learning more closely to biology [69]–[71]. Future work will nevertheless try to use alternative solutions for learning of the synaptic weights.

Unfortunately we were not able to identify meaningful intermediate behavioral strategies while the model was still learning. It would be interesting to compare the behavior of the RL agent with recent empirical and theoretical work on the learning of cue integration, which suggest potentially different behavior such as calibration of a less reliable modality by a more reliable one [17], [28], [72], [73] or using the modalities alternatingly [18] maybe according to the so called race model [19], [20]. The modality providing the basis for calibration could depend on the relative reliabilities, could be innately determined or chosen randomly. Consistent with the first option are results showing that even unisensory performance in certain non–visual tasks can be worse in early blind compared to sighted children [72].

To conclude, the RL algorithm with function approximation was capable of learning near optimal performance in the Bayesian sense for both cue integration and causal inference tasks (consistent with previous results with tabular RL [48]). Importantly, despite not performing explicit computations with uncertainties, the reinforcement learner successfully changed actions depending on the uncertainty in the stimulus. Considerable evidence about the neural basis of such algorithms makes this approach appealing. Furthermore, it gives a direct way of accommodating learning of cue integration and causal inference over developmental timescales. Thus, even if RL algorithms may not be the only mechanisms underlying the human development of cue integration and causal inference they could definitely contribute to their development.

## Methods

### Task Setup

In our task each trial consists of the presentation of two stimuli in the visual and auditory modalities. These stimuli either originate from a single common source (Fig. 1 left) for the auditory and visual cue or from two separate sources/objects (Fig. 1 right). A position 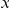 along the spatial dimension is chosen from a uniform distribution for each object in the scene (but results if, e.g., visual prior is Gaussian around the central region are not different – Table 1 1C). In the two objects case we call their positions 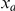 (for the object that emits only an auditory signal) and 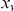 (for the object that emits only a visual signal). Space is discretized to 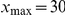 positions for ease of computation. The received sensory signals are noisy versions of the true source locations. We use additive noise with normal distributions with zero mean and variances 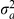 and 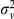. Note that the RL model is also able to deal with noise from different distributions since we do not implement the learner based on a fixed distribution. See Table 1 2B for a setup with auditory and visual noise drawn from a logistic distribution with median 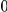. This noise is thought as being of sensory and/or environmental origin, e.g. background noise, neuronal firing stochasticities and tuning densities. Usually the variance of the auditory estimate is set larger than the visual one, in accordance with psychophysical observations for spatial tasks [74]. We call this noisy signal position 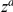 and 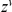 respectively. If the noise makes a signal fall outside of the spatial range, the stimulus is treated as not present, thus resulting in a unisensory training trial. An important implication of this setting is that the structure of observations is the same for both possible underlying generative models.

We use two slightly different versions of this task. In the single output task the learner has to orient towards a single location. That means in the case of two objects the reward only depends on the distance to the object closest to the estimated position. In the two outputs task it is required to orient towards both the visual and the auditory positions of their respective cause. In case of a common cause this should result in both estimates being equal. There are separate rewards for the visual and auditory action. The inputs were the same for both experiments.

### Reinforcement Learning Model

An approximation of the function relating state-action pairs to predicted reward is learned. A three-layered neural network (see Fig. S1) is set up with an input unit for each position in every modality (here 60 input neurons). It should be mentioned that the yet unsolved problem of limited scalability of RL approaches for very large numbers of inputs, does also apply to our model. The input neurons 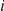 are all-to-all connected with weights 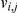 to neurons 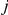 in the hidden layer (here j = 0…29). Stimulus locations 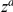 and 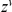 are represented by the population activity of these input neurons (see e.g. [75], [76] for biological examples of population codes) in each modality separately (the first half of the neuron coding for the auditory input, the second for the visual one).

Each input neuron has a Gaussian receptive field, centered on position 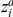 or respectively 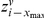 for 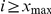. The variance of these Gaussians is in the order of the noise of the input stimuli. Overlapping receptive fields of the input neurons simply help the network to discover a spatial relationship between the possible input positions. We also tested the framework with simple binary input units and found no difference in the final results besides an increase in learning time (see Table 1 1B).

A sigmoidal transfer function on the sum of the weighted inputs 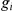 produces the activation 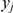 of the hidden neurons. These are again fully connected to the output neurons 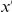 with weights 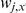. For every action 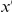 there is one output unit, with its activation 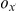 , given by the weighted sum of the hidden layer activity, representing an approximation of the appropriate Q-value. All weights are drawn from a uniform distribution, the 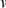
's between 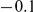 and 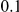, the 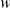
's between 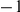 and 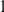.

Based on the network's outputs the learner chooses one of the available actions. This is done with the softmax function:
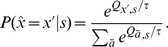
(1)This probabilistic action selection rule chooses an action 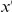 with a probability proportional to the relative predicted reward 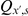 for that action, given state 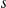. We start with a high temperature parameter 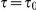, so that the learner chooses his actions only weakly influenced by the initial reward expectations. 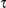 then decreases exponentially with learning time (with 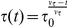), passing 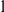 after a given number of steps 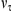. At smaller values of 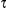 the selection favors more and more the action with highest expected reward, thus exploiting the environment.

After performing the selected action 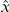, the learner receives the true reward 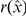. We use a reward function that is maximal if 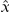 equals the true object position 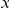, decaying quadratically with increasing distance within a surrounding area (with radius 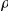) and zero otherwise.

For single output:
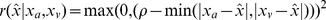
(2)For two outputs:
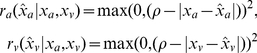
(3)If only one object is present, the two position are equal, i.e. 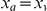. In the experiments shown above we used 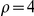. Changes of of 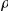 other than setting it to zero (only rewarding correct actions) only have an impact on the learning time. We also tested the model with an asymmetric reward function, where a correct visual action would only provide half the reward of a correct auditory action (results see Table 1 2C).

Based on the true reward, the Q-values for the particular state-action pair will be updated proportional to the difference between prediction 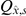 and 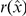. This difference can be seen as a temporal difference (TD) error for a single timestep. TD learning in general uses discounted future rewards for computing the prediction error: The Q-value function will not only represent the expected reward of a single state–action pair, but also include possible future rewards that are expected from the new state. In the present work the learner has to only perform a single action per trial and receives only immediate reward.

To minimize the TD error we use gradient descent to change the weights of the neural network by 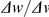 [77] with:
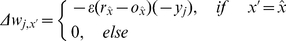
(4)

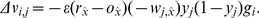
(5)

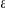
 is an exponentially decreasing learning rate: 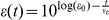, with 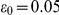 and 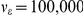. The results did not change when using an alternative function for the learning rate, 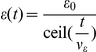, with 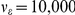.

### Bayesian Observer Models

We compare the performance of our model with that of four different Bayesian observers, inferring the position of the object given the input and the generating model 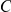 (Fig. 1 bottom). With Bayes' theorem and the assumption that the noise of different modalities is independent we can write the posterior probability as:
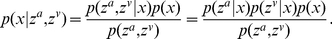
(6)where the last equality is only valid if the two cues are conditionally independent given their cause. The likelihoods 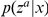 and 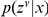 include all information available from the input. The reliability of a cue is inversely proportional to the standard deviation of this distribution. In the experiments reported in this paper the prior 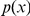 is always uniformly distributed. Other priors were used in simulations not shown, and the RL algorithm was able to adjust to these and still perform close to the Bayesian predictions. Since we are interested in the performance of the model in terms of reward, actions are not chosen only based on the posterior probabilities, but on the utility function 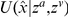, which additionally takes into account the expected reward 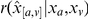 (we write 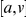 to cover both the one and two output case) for a given action (see below). The use of different utility functions can accommodate different tasks in a very direct way and makes the behavioral goal explicit.

The Bayesian observers used here differ in the way they handle the two different possible generative models (one vs. two causes; Fig. 1 bottom). Model Averaging (MA) uses a utility function that is a weighted average of the inference results of each model. The weights are determined by the probability for one versus two objects 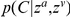. This probability can again be computed from known distributions using Bayes formula similar to (6).
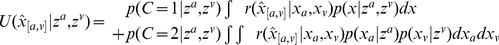
(7)


.Model selection (MS) in contrast uses only the utility function of the most probable model.
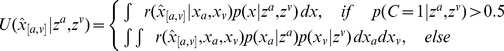
(8)


We use a uniform prior over the number of objects in the scene (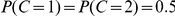). Results of additional simulations not shown here lead to similar results for asymmetric distributions.

We also consider two observers that only do inference on one model, ignoring the second one – one always integrates the information (AI) and the other always treats the inputs as independent (NI). The utility functions of all observer models are computed by numerical integration. For a given input we choose the action with maximum utility.

Another possible observer model would compute the same probability distributions as in MS and MA, but then select stochastically from them instead of choosing the maximum. Such a behavior is often called Probability Matching (PM). In our case it could be used in two ways. A recent paper proposed PM at the level of causal inference [15], an action will be chosen according to one of the generating models with the probability for that model to be the underlying cause (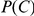). Because this is an intermediate between MA and MS we only consider it when computing the 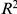, where we distinguish between those. The second possibility would be to use PM for the action selection step, which was found in various studies to be a strategy employed by human observers in certain tasks [78], [79]. This is actually implicitly assumed in our model by using the softmax function to pick the action, thereby we do not include this option in our analysis.

## Supporting Information

Figure S1 **Sketch of the neural network used for approximation of the Q-value function.** Setup for the two–step orienting task, the setup for the simple orienting task differs only in that the network has only half as many output neurons, since only a single action is required.
(EPS)

Figure S2
**PSEs of the model for the 2-AFC task.** Plot of the PSEs for each of 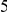 repetitions of training and 2-AFC testing with different values for the variance of the visual noise 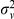. For all trials 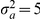. In the test trials the visual signal of the standard was always at 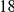, the auditory at 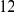. PSEs of the RL model (black crosses) and Bayesian integration (red circles).(EPS)

Figure S3

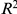
 **values of different observers for the responses of the RL model for all inputs.** Same Plot as Fig. 6 but using inputs from the full space. The black and gray bars show the results for the auditory and the visual output for 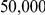 trials. Mean over 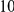 training sessions with 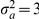, 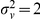, errorbars show standard deviation.(EPS)

Figure S4
**Input weights of the NN for the foveation setup.** Input weights 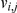 to representative hidden neurons. The left plot shows the weights only from visual input neurons (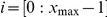), the right only from the auditory input neurons (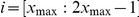).(EPS)
